# Summary of World Café Discussions Table 1: Assessment

**DOI:** 10.25646/6507

**Published:** 2020-06-04

**Authors:** Angela Fehr, Giselle Sarganas

**Affiliations:** 1 Robert Koch Institute, Berlin, Centre for International Health Protection; 2 Robert Koch Institute, Berlin, Department of Epidemiology and Health Monitoring

Health information is an essential component of the public health action cycle. If measures to promote health, to prevent disease and to deliver health care are to respond to real needs, they need to be guided by proper health information. Health information is needed to assess existing public health or policy measures over time, thereby supporting and evaluating an existing agenda (agenda keeping). At the same time, health information may point to emerging issues requiring new political and/or scientific attention (agenda setting).

Proceeding from this idea that prioritization in health information can have different objectives, we asked the following question: ‘Which tools/methods for systematic evidence assessment does the Robert Koch Institute as a national public health institute need to identify and prioritize public health topics?’

The discussion focused on two aspects: Firstly, who shall be involved in prioritization? Secondly, what processes are needed for prioritization? In terms of involvement, the experts pointed to the need for broad stakeholder involvement and for a building and maintaining cross-sectoral networks. Qualification of experts in these networks was deemed important; however, it was underlined that a balanced mix of experts and generalists may broaden horizons and ensures consideration of new perspectives.

Establishing processes for prioritization was considered to be challenging. Discourse analysis was mentioned as a useful tool to identify emerging topics. Furthermore, to ensure a proper evidence base, participants recommended to base prioritization on burden of disease and social inequality data. At the same time, it was pointed out that, in practical terms, priorities are not always data-based, but may, for example, follow a donor’s agenda, especially in low-income settings.

Participants underlined that, when discussing prioritization, a distinction has to be made between immediate and longer-term needs for action, as in reacting to outbreaks versus combatting non-communicable diseases. Patience and persistence were named as essential traits in advocating topics that do not pose immediate public health threats. Experience had shown that, in those cases, it may even be prudent to let an opportunity for action pass by and wait for another window to open. With a view to continuously working with stakeholders to identify relevant public health issues, a platform such as the German ‘Future Forum Public Health’ (Zukunftsforum Public Health) was considered useful. Finally, participants underlined that de-prioritization of public health topics, e.g. in case of lack of feasibility, requires equal attention and systematic approaches.

